# Traumatic subconjunctival lens dislocation

**DOI:** 10.1002/ccr3.8098

**Published:** 2023-10-19

**Authors:** Mehrdad Motamed Shariati, Seyedeh Maryam Hosseini, Zeinab Hashemi Javaheri

**Affiliations:** ^1^ Eye Research Center Mashhad University of Medical Sciences Mashhad Iran

**Keywords:** crystallin lens dislocation, ocular trauma, open globe injury

## Abstract

Traumatic crystallin lens dislocation is a common complication following open globe injuries. Wound repair, lensectomy, and prophylactic antibiotic administration are essentials in the management.

## 1 IMAGE DESCRIPTION

**FIGURE 1:**
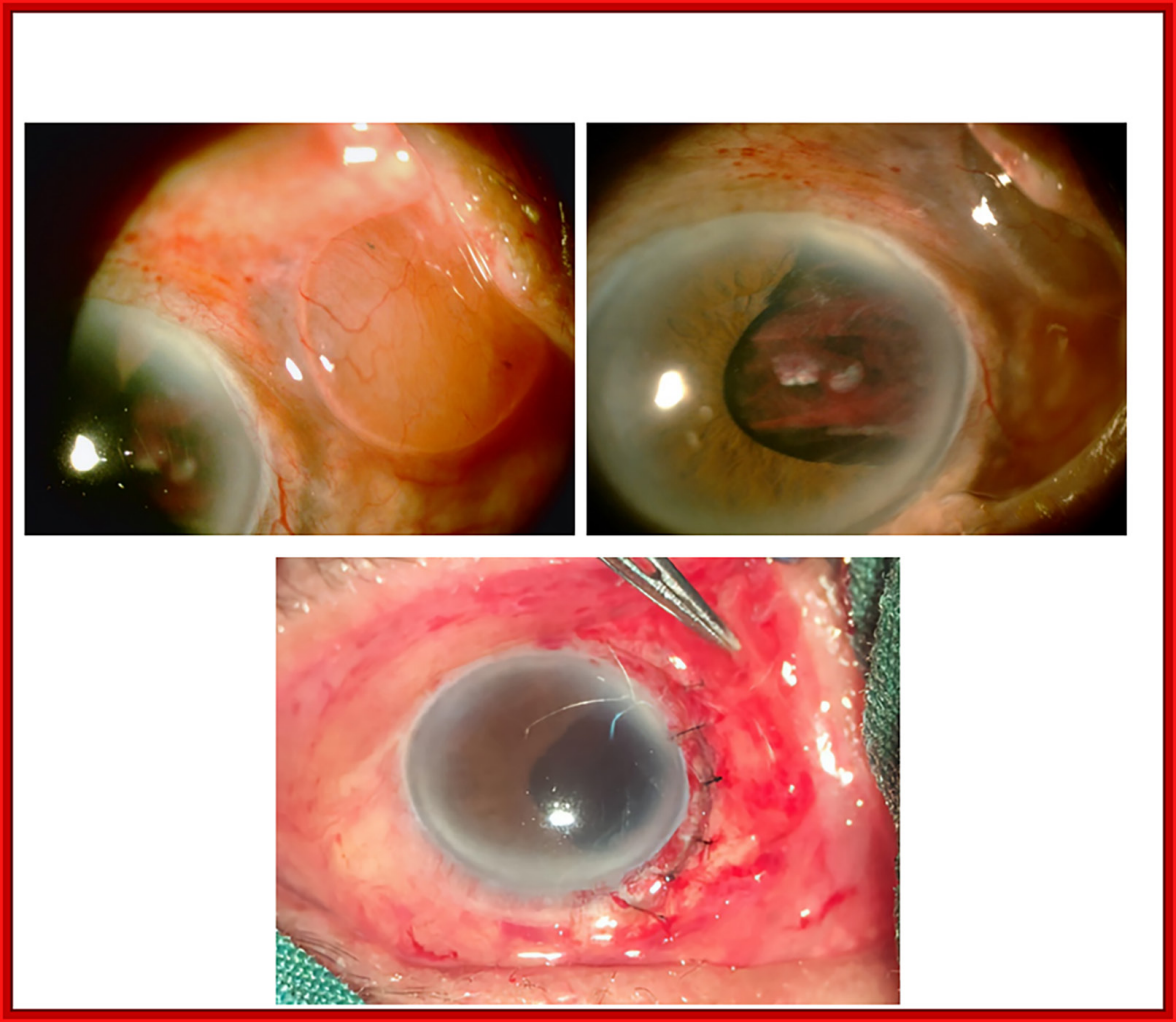
Open globe injury with crystallin lens dislocation to the subconjunctival space.

A 75‐year‐old man with a history of ocular blunt trauma of the right eye (RE) 2 days ago was referred to our clinic with visual loss and pain. In the ophthalmic examination, we found traumatic scleral rupture and subconjunctival crystallin lens dislocation (Figure 1, upper row). The best corrected visual acuity (BCVA) was hand motion detection for the RE and 20/20 for the left eye (LE). The relative afferent pupillary defect (RAPD) was negative. Fundus examination and intraocular pressure revealed no significant abnormalities in the left eye. The patient underwent surgical repair of the scleral wound, crystallin lens extraction, and wound vitrectomy (Figure 1, lower row). The best corrected visual acuity was 3/10 2 weeks after the primary repair surgery. The patient is a candidate for iridoplasty and secondary intraocular lens implementation.

## 2 DISCUSSION

**FIGURE 2:**
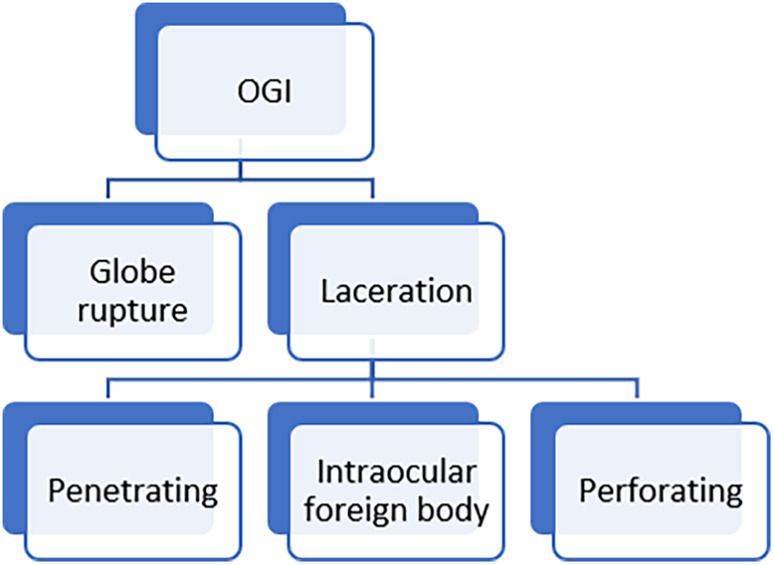
OGI classification.

Open globe injuries (OGI) are defined as full‐thickness defects of the eyewall and are among the major public health problems in developing countries.^1^ Regarding the severity of the injury, a wide range of complications could lead to permanent vision loss. Traumatic optic neuropathy, traumatic maculopathy, choroidal rupture, and retinal detachment are common causes of blindness following OGI.^2^ Depending on the mechanism of injury, traumatic open globe injuries are classified as globe rupture and laceration (Figure 2). Globe rupture occurs following blunt trauma to the eye wall, while lacerations are due to sharp trauma.

### 2.1 Clinical approach for a patient with OGI

**FIGURE 3:**
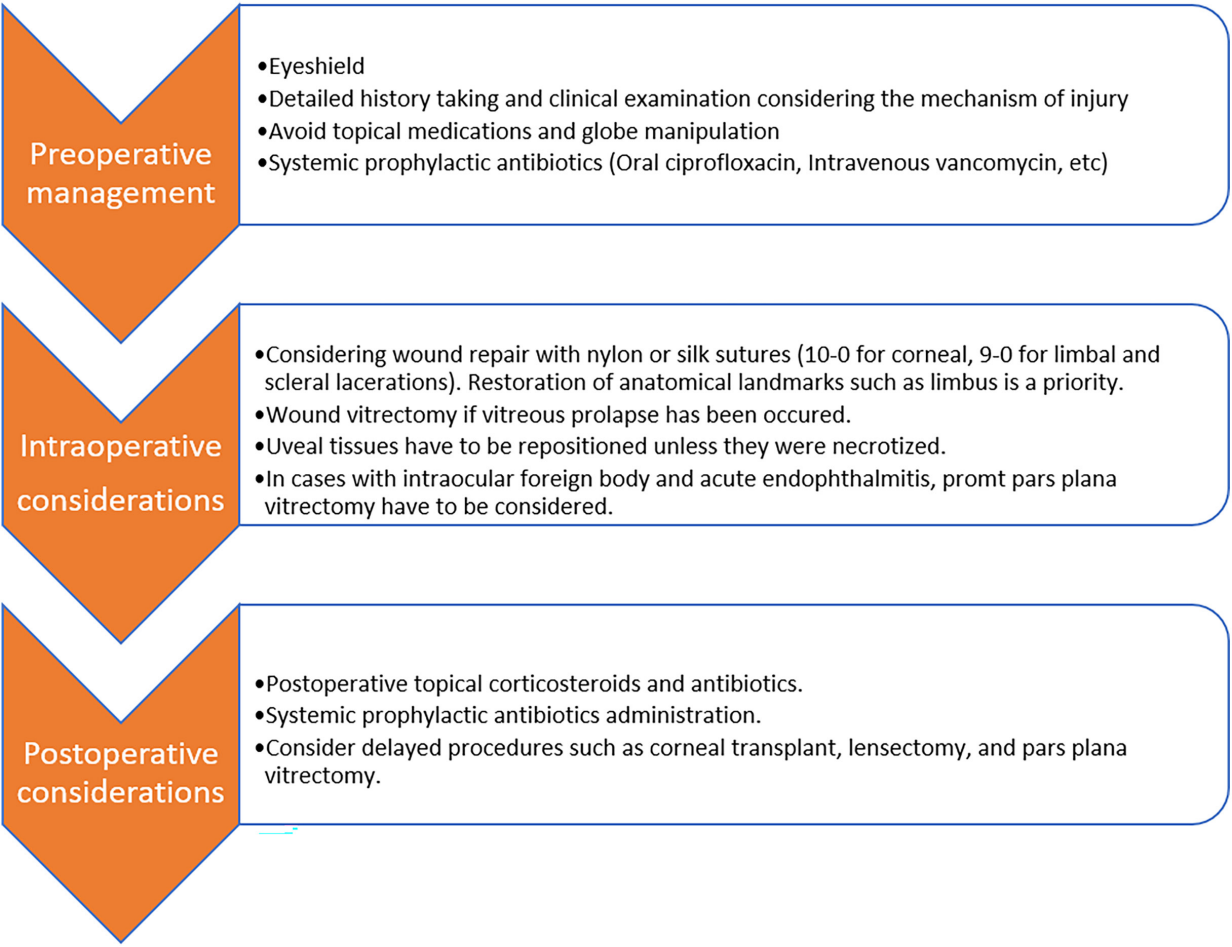
Clinical management for a patient with OGI.

We summarized the management approach for a patient with OGI in Figure 3.^3^

## AUTHOR CONTRIBUTIONS

**Mehrdad Motamed Shariati:** Conceptualization; investigation; project administration; supervision; writing – original draft; writing – review and editing. **Seyedeh Maryam Hosseini:** Data curation; supervision; writing – review and editing. **Zeinab Hashemi Javaheri:** Data curation; investigation.

## FUNDING INFORMATION

The authors received no funding.

## CONFLICT OF INTEREST STATEMENT

The authors declare that they have no competing interests.

## CONSENT

Written informed consent was obtained from the patient for the publication of this clinical image report.

## Data Availability

The data that support the findings of this study are available on request from the corresponding author. The data are not publicly available due to privacy or ethical restrictions.
